# Breast cancer risk among women with psychiatric admission with affective or neurotic disorders: a nationwide cohort study in Denmark

**DOI:** 10.1038/sj.bjc.6690785

**Published:** 1999-11

**Authors:** K Hjerl, E W Andersen, N Keiding, A Sawitz, J H Olsen, P B Mortensen, T Jørgensen

**Affiliations:** Department of Surgery K, Copenhagen University Hospital Corporation, Bispebjerg Hospital, Copenhagen NV DK-2400, Denmark; Departments of Biostatistics, Social Medicine and Psychosocial Health, Institute of Public Health, University of Copenhagen, 3 Blegdamsvej, Copenhagen N DK-2200, Denmark; Institute of Cancer Epidemiology, Danish Cancer Society, 49 Strandboulevarden, Copenhagen Ø DK-2100, Denmark; Institute for Basic Psychiatric Research, Department of Psychiatric Demography, University of Aarhus, Psychiatric Hospital of Aarhus, Risskov DK-8240, Denmark; Centre of Preventive Medicine, Medical Department C/F, Glostrup University Hospital, 57 Nordre Ringvej, Glostrup, DK-2600 Denmark

**Keywords:** neoplasm breast, aetiology, affective disorder, neurotic disorders, alcohol abuse, non-specified abuse

## Abstract

There is a considerable interest in the possible relationship between psychosocial factors and the onset of breast cancer. This cohort study was based upon two nationwide and population-based central registers: The Danish Psychiatric Central Register, which contains all cases of psychiatric admissions, and The Danish Cancer Registry, which contains all cases of cancer. The register-linkage was accomplished by using a personal identification number. The study population comprised all women admitted to psychiatric departments or psychiatric hospitals in Denmark between 1969 and 1993 with an affective or a neurotic disorder. Overall, 66 648 women comprising 199 910 admissions and 775 522 person-years were included. The incidence of breast cancer in the cohort was compared with the national breast cancer incidence rates adjusted for age and calendar time. In all, 1270 women with affective or neurotic disorders developed breast cancer subsequent to the first admission as compared with the 1242 women expected, standardized incidence ratio (SIR) = 1.02 (95% confidence interval 0.97–1.08). None of the hypothetical risk factors: type of diagnosis, age or calendar period at cohort entry, age at breast cancer, alcohol abuse, alcohol/drug abuse without further specification, total number of admissions, total length of admissions, or time from first admission showed a statistically significant effect on the relative risk of breast cancer. We found no support for the hypothesis that women admitted to a psychiatric department with an affective or a neurotic disorder subsequently have an increased risk of breast cancer. © 1999 Cancer Research Campaign


British Journal of Cancer (1999) 81(5), 907–911
© 1999 Cancer Research Campaign
Article no. bjoc.1999.0785

### Breast cancer risk among women with psychiatric admission with affective or neurotic disorders: a nationwide cohort study in Denmark

**K Hjerl[1], EW Andersen[2], N Keiding[2], A Sawitz[3], JH Olsen[4], PB Mortensen[5] and T Jørgensen[6]**

[1]Department of Surgery K, Copenhagen University Hospital Corporation, Bispebjerg Hospital, DK-2400 Copenhagen NV, Denmark; Departments of [2]Biostatistics and [3]Social Medicine and Psychosocial Health, Institute of Public Health, University of Copenhagen, 3 Blegdamsvej, DK-2200 Copenhagen N, Denmark; [4]Institute of Cancer Epidemiology, Danish Cancer Society, 49 Strandboulevarden, DK-2100 Copenhagen Ø, Denmark; [5]Institute for Basic Psychiatric Research, Department of Psychiatric Demography, University of Aarhus, Psychiatric Hospital of Aarhus, DK-8240 Risskov, Denmark; [6]Centre of Preventive Medicine, Medical Department C/F, Glostrup University Hospital, 57 Nordre Ringvej, DK-2600 Glostrup, Denmark

**Summary** There is a considerable interest in the possible relationship between psychosocial factors and the onset of breast cancer. This cohort study was based upon two nationwide and population-based central registers: The Danish Psychiatric Central Register, which contains all cases of psychiatric admissions, and The Danish Cancer Registry, which contains all cases of cancer. The register-linkage was accomplished by using a personal identification number. The study population comprised all women admitted to psychiatric departments or psychiatric hospitals in Denmark between 1969 and 1993 with an affective or a neurotic disorder. Overall, 66 648 women comprising 199 910 admissions and 775 522 person-years were included. The incidence of breast cancer in the cohort was compared with the national breast cancer incidence rates adjusted for age and calendar time. In all, 1270 women with affective or neurotic disorders developed breast cancer subsequent to the first admission as compared with the 1242 women expected, standardized incidence ratio (SIR) = 1.02 (95% confidence interval 0.97–1.08). None of the hypothetical risk factors: type of diagnosis, age or calendar period at cohort entry, age at breast cancer, alcohol abuse, alcohol/drug abuse without further specification, total number of admissions, total length of admissions, or time from first admission showed a statistically significant effect on the relative risk of breast cancer. We found no support for the hypothesis that women admitted to a psychiatric department with an affective or a neurotic disorder subsequently have an increased risk of breast cancer. © 1999 Cancer Research Campaign

**Keywords:** neoplasm breast; aetiology; affective disorder; neurotic disorders; alcohol abuse; non-specified abuse

Breast cancer is the most common cancer among women in the Western world, with a cumulative lifetime risk in Denmark of 1 in 12 (Danish National Board of Health, 1997). The number and activity of natural killer cells have been reported as significantly affected in depressed patients (Irwin et al, 1987; Maes et al, 1992; Schleifer al, 1996; Andersen et al, 1998; Cohen et al, 1998), which could be the possible mechanism for depression as a risk factor for breast cancer.

At least two well-conducted case–control studies (Chen et al, 1995; Ginsberg et al, 1996) have demonstrated an association between major negative life events and breast cancer, although other large-scale follow-up studies have been negative (Jones et al, 1984; Ewertz, 1986; Kvikstad et al, 1994; Roberts et al, 1996; Johansen and Olsen, 1997). Clinical affective and neurotic disorders were not assessed in the studies of adverse life events.

The existence of a cancer-prone personality with depressive and neurotic symptoms has been proposed mainly in case-reports and studies with cross-sectional design; case–control (Bleiker et al, 1996) or follow-up studies (Hagnell, 1966) have been sparse.

*Received 13 January 1999*
*Revised 9 March 1999*
*Accepted 11 March 1999*

*Correspondence to:* K Hjerl, Department of Psychiatry E, Bispebjerg University Hospital, 23 Bispebjerg Bakke, DK 2400 Copenhagen, Denmark

In cohort studies of women with depressive symptoms (Zonderman et al, 1989), neurotic disorders (Zilber et al, 1989) and affective disorders (Weeke and Vaeth, 1986), the standardized mortality ratio (SMR) of cancer in general was no different from that in the normal population of women. In a cohort study, no significant difference in breast cancer risk was reported among women who rated themselves as depressive and those who did not (Hahn and Petitti, 1988).

The aim of the present study was to test the hypothesis that clinical affective or neurotic disorders are risk factors for breast cancer. We also wanted to test if the co-variables: age at first depressive or neurotic episode, duration of period from first episode, type of diagnosis, calendar period at first episode, alcohol abuse, or alcohol/drug non-specified abuse had effects as determinants. In addition, we wanted to test whether a long psychiatric in-patient treatment period, as a proxy variable for long periods of supervised treatment with psychotropics, was a risk factor for breast cancer development.

### PATIENTS AND METHODS

The study population comprised women who had been admitted to psychiatric departments in general hospitals or psychiatric hospitals in Denmark with the first admission with a affective or a neurotic disorder at an age of more than 15 during the period 1 April 1969 to 31 December 1993. Relevant information was drawn from the Danish Psychiatric Central Register (Munk-Jørgensen and

**Table 1** Five ordinal types of affective and neurotic diagnoses

| Types | Diagnoses[a] | Codes |
|---|---|---|
| 1. Bipolar | Manic-depressive psychosis, manic type | 296.19 |
| | Manic-depressive psychosis, circular type | 296.39 |
| 2. Unipolar | Involutional melancholia | 296.09 |
| | Manic-depressive psychosis, depressed type | 296.29 |
| | Manic-depressive psychosis, other | 296.89 |
| | Manic-depressive psychosis, unspecified | 296.99 |
| 3. Reactive | Reactive depressive psychosis | 298.09 |
| 4. Dysthymic | Affective (cyclothymic) personality disorder | 301.19 |
| | Neurotic depression | 300.49 |
| 5. Neurotic | All other neuroses | 300.00–300.99 (except 300.49) |
| | Other personality disorders | 301.81 |

[a] Modified Danish revision of the eighth revision of the International Classification of Diseases.

Mortensen, 1997), which since 1 April 1969 has been computerized and updated to include all admissions to all psychiatric departments and hospitals in the country. Each admission record includes the personal identification number, date of admission, date of discharge, one main discharge diagnosis and up to three auxiliary discharge diagnoses. The personal identification number, which is unique to every Danish citizen, incorporates sex and date of birth, and permits accurate linkage of information between registries. Discharge diagnoses were recorded according to the International Classification of Diseases, 8th Revision (ICD-8) (WHO, 1967).

For the purpose of this study, the affective and neurotic diagnoses were categorized in five types (Table 1). Types 1, 2 and 3 represented psychotic affective disorders, type 4 represented non-psychotics and type 5 represented neurotic disorders. Cohort entry was defined as the date of first admission with an affective or neurotic disorder, and the patient was categorized according to the type of diagnosis presented during this admission. If re-admitted to a psychiatric department with a diagnosis belonging to another type with a lower number, the patient was re-categorized from that date and onwards with the new lower number; however, if re-admitted with the same type of diagnosis or another type with a higher number, the patient's categorization remained unchanged. Diagnostic information of alcohol abuse (ICD-8 code 303) and diagnostic information of alcohol/drug non-specified abuse (code 304) was retained together with first date of diagnosis. If re-admitted with a diagnosis of schizophrenia (code 295), the patient was censored from the first date of admission with this diagnosis.

The study population was linked to the files of the Danish Cancer Registry, which collects information about all patients with cancer in Denmark (Storm et al, 1996). The period of follow-up for cancer occurrence was taken from the date of first admission with an affective or neurotic disorder (cohort entry) until the date of death (obtained from the national mortality files), date of diagnosis of schizophrenia, or 31 December 1993, whichever occurred first. Of the 67 285 women 15 years or older, diagnosed with an affective or neurotic disorder, 637 (0.1%) had been diagnosed with breast cancer and entered onto the Cancer Registry before date of cohort entry. They were excluded from this study, leaving 66 648 women for evaluation (Table 2).

**Table 2** Descriptive characteristics of 66 648 women with affective or neurotic disorders

| Characteristic | Number | Per cent |
|---|---|---|
| Entire study cohort | 66 648 | 100 |
| Age at cohort entry | | |
| < 30 | 13 105 | 19.7 |
| 30–39 | 14 488 | 21.7 |
| 40–49 | 13 155 | 19.7 |
| 50–59 | 11 254 | 16.9 |
| 60–69 | 8 170 | 12.2 |
| 70–79 | 5 084 | 7.7 |
| > 80 | 1 392 | 2.1 |
| Year of cohort entry | | |
| 1969–1973 | 15 305 | 23.0 |
| 1974–1978 | 17 323 | 26.0 |
| 1979–1983 | 13 840 | 20.0 |
| 1984–1988 | 11 136 | 17.4 |
| 1989–1993 | 9 044 | 13.6 |
| Initial type of diagnosis | | |
| Affective | | |
| Bipolar | 2 417 | 3.6 |
| Unipolar | 16 289 | 24.4 |
| Reactive | 10 222 | 15.3 |
| Dysthymic | 9 608 | 14.4 |
| Neurotic | 28 112 | 42.2 |
| Place of residence at cohort entry | | |
| Capital | 11 930 | 17.9 |
| Suburbs | 8 958 | 13.4 |
| Provincial towns | 26 794 | 40.2 |
| Rural areas | 18 961 | 28.5 |
| Missing information | 5 | |
| Total number of admissions | | |
| 1 | 28 418 | 42.6 |
| 2–5 | 28 556 | 42.8 |
| 6–10 | 6 379 | 9.6 |
| ≥ 11 | 3 295 | 4.9 |
| Diagnosis of abuse | | |
| Yes | | |
| Alcohol | 5 355 | 8.0 |
| Alcohol/drug | 8 258 | 12.4 |
| No | 53 035 | 87.6 |

### Statistical analysis

The expected numbers of cancers were calculated by multiplying the number of person-years of cohort members by the breast cancer incidence for Danish women in 5-year age groups and calendar periods of observation. Tests of significance and confidence intervals for the standardized incidence ratio (SIR), taken as the ratio of observed to expected breast cancers, were based on standard log-linear models, multiplicative intensity models, often termed 'Poisson regression' (Clayton and Hills, 1993).

© 1999 Cancer Research Campaign

**Table 3** Observed number (Obs) and standardised incidence ratio (SIR) for breast cancer in 66 648 women with affective or neurotic disorders by type of diagnosis, use of treatment as in-patients in psychiatric departments, personal characteristics and diagnoses of abuse

| Characteristic | Number of person-years | Obs. | SIR | 95% CI |
|---|---|---|---|---|
| Entire study cohort | 775 522 | 1 270 | 1.02 | 0.97–1.08 |
| Age at cohort entry | | | | |
| < 30 | 65 052 | 2 | 0.72 | 0.12–2.22 |
| 30–39 | 149 319 | 57 | 0.91 | 0.69–1.17 |
| 40–49 | 178 385 | 271 | 1.01 | 0.90–1.14 |
| 50–59 | 156 796 | 318 | 0.99 | 0.88–1.10 |
| 60–69 | 125 997 | 315 | 1.06 | 0.95–1.18 |
| 70–79 | 75 281 | 220 | 1.04 | 0.91–1.19 |
| ≥ 80 | 24 692 | 87 | 1.10 | 0.88–1.34 |
| Year at cohort entry | | | | |
| 1969–1973 | 31 753 | 33 | 1.06 | 0.74–1.47 |
| 1974–1978 | 112 124 | 127 | 0.95 | 0.79–1.12 |
| 1979–1983 | 177 593 | 254 | 1.02 | 0.90–1.15 |
| 1984–1988 | 217 482 | 362 | 1.00 | 0.90–1.11 |
| 1989–1993 | 236 569 | 494 | 1.06 | 0.97–1.16 |
| Type of diagnosis | | | | |
| Bipolar | 38 709 | 56 | 0.90 | 0.69–1.16 |
| Unipolar | 190 264 | 377 | 0.99 | 0.90–1.10 |
| Reactive | 115 551 | 193 | 1.02 | 0.88–1.17 |
| Dysthymic | 130 012 | 228 | 1.10 | 0.95–1.25 |
| Neurotic | 300 986 | 416 | 1.03 | 0.94–1.14 |
| Number of admissions | | | | |
| 1 | 392 939 | 632 | 1.02 | 0.94–1.10 |
| 2 | 159 544 | 270 | 1.04 | 0.93–1.17 |
| 3 | 78 411 | 143 | 1.11 | 0.94–1.31 |
| 4–5 | 70 959 | 116 | 0.99 | 0.82–1.18 |
| 6–10 | 52 860 | 79 | 0.91 | 0.72–1.12 |
| ≥ 11 | 20 809 | 30 | 0.93 | 0.64–1.31 |
| Length of admissions | | | | |
| < 1 year | 737 568 | 1 215 | 1.03 | 0.98–1.09 |
| 1–2 years | 26 152 | 40 | 0.91 | 0.66–1.22 |
| > 2 years | 11 801 | 15 | 0.69 | 0.40–1.10 |
| Period at follow-up (years) | | | | |
| 0–4 | 291 547 | 383 | 1.00 | 0.91–1.11 |
| 5–9 | 223 626 | 345 | 0.99 | 0.89–1.10 |
| 10–19 | 239 089 | 492 | 1.06 | 0.97–1.16 |
| ≥ 20 | 21 260 | 50 | 1.01 | 0.76–1.32 |
| Diagnosis of abuse | | | | |
| Alcohol | 85 758 | 140 | 1.12 | 0.94–1.30 |
| Alcohol/drug | 130 420 | 206 | 1.04 | 0.91–1.19 |

### RESULTS

For the 66 648 cohort members, 775 522 person-years of follow-up were accrued, median follow-up was 11.6 years (range 0–25 years) and median age at first admission 44 years (Table 2). The cohort experienced a total of 199 910 psychiatric admissions, of which 32 474 entailed a re-categorization of type of diagnosis compared to that of previous admissions.

At cohort entry, a total of 38 536 patients (58%) were registered with an affective disorder and 28 112 (42%) with a neurotic disorder. During follow-up, 28 418 patients (42%) were admitted only once. Psychiatric co-morbidity with alcohol abuse or alcohol/drug abuse was registered in 5355 (8%) and 8258 (12%) of the subjects respectively. Follow-up was suspended because of a diagnosis of schizophrenia in 1240 patients (2%) and because of death in 16 435 patients (25%).

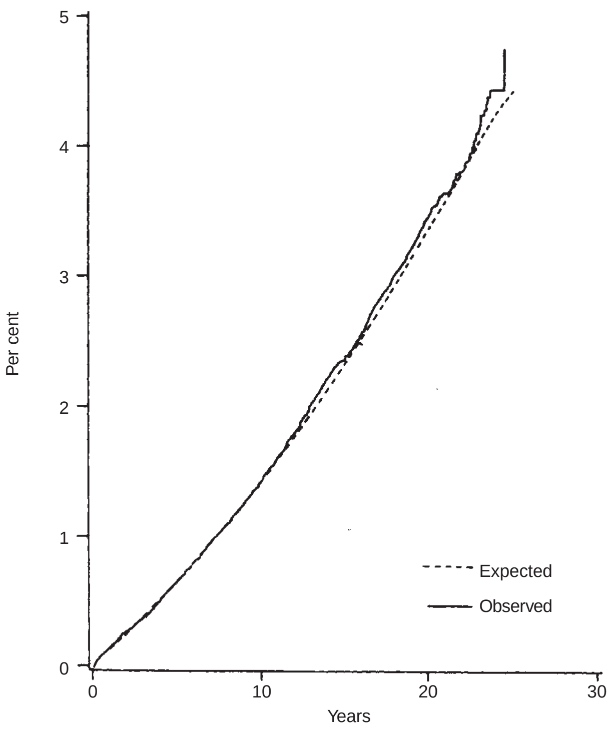

**Figure 1** Observed and expected risk of primary invasive breast cancer by time from first psychiatric admission with affective or neurotic disorder

Overall, 1270 breast cancers were observed, and 1241 were expected, yielding a SIR of 1.02 (95% confidence interval (CI) 0.97–1.08) (Table 3). The diagnoses of primary invasive breast cancer were confirmed histopathologically in 97.5% of cases.

Neither age nor year at cohort entry showed any remarkable pattern or significant findings in any of the subgroup analyses (Table 3). The cumulative risk of women with affective and neurotic disorder for breast cancer, by time from date of first admission, was similar to the expected cumulative risk, which was based on the national incidence rate of female breast cancer (Figure 1). Type of diagnosis showed no statistical significant difference, although the risk was slightly higher in the subgroup of women with dysthymia (SIR of 1.10) (95% CI 0.95–1.25). Women with a diagnosis of alcohol abuse had an increased risk (SIR of 1.12) (95% CI 0.94–1.30), but the difference was not significant. There is no evidence of a positive relationship between length of admission and breast cancer risk.

### DISCUSSION

We found no support for the hypothesis of an increased risk of breast cancer among women discharged from psychiatric departments or hospitals with affective or neurotic disorders, and none of the co-variables – age at cohort entry, calendar year at cohort entry, type of diagnosis, number of admissions, length of admissions, period of follow-up, alcohol abuse or alcohol/drug abuse – had any appreciable effect as determinants. The large data set implied the narrow confidence intervals, which exclude the possibility that this result is a coincidence.

Large well-performed cohort studies of affective or neurotic disorders as risk factors for breast cancer are sparse. In a cohort

© 1999 Cancer Research Campaign study of women, self-rated with Minnesota Multiphasic Personality Inventory, 9% were depressive at baseline. During 10 years of follow-up 117 women developed breast cancer, and no significant difference was reported among women who rated themselves as depressive and those who did not (Hahn and Petitti, 1988). This study was hampered by a highly selected study population and most women were under age of 50 years. In a Danish cohort study of 1325 women discharged from psychiatric departments and hospitals with bipolar or unipolar manic-depressive disorders the standardized mortality ratio of cancer in general was not different from the rate in the general population, but the specific standardized mortality ratio of breast cancer was not assessed (Weeke and Vaeth, 1986). The present study represents the first large cohort study of affective and neurotic disorders in relation to breast cancer.

Our data from the nationwide registries have high validity (Storm, 1977; Kessing, 1998) and the same diagnostic classification system has been used during the whole study period (WHO, 1967). The information about cancer cases in Denmark is almost complete (Storm, 1988; Rostgaard and Lynge, 1997), and we have no reason to believe that this cohort had a higher emigration rate than that of the general population of females, i.e. 0.5% per year (Statistics Denmark, 1998). In addition, we supposed that the median follow-up period of 11.6 years was sufficient for aetiological cancer research.

Being a register-study all data have been originally collected for other purposes, and thus the available data do not cover risk factors for breast cancer, other than age and calendar period, which were included in all analyses. There is no evidence that distribution of well-established risk factors for breast cancer among Danish women admitted to psychiatric departments with affective or neurotic disorders are different from the general population of women, except for reduced fertility rate (Ødegaard, 1980), which could have implied a slight overestimation. Our cohort did not comprise all Danish women with depressive or neurotic disorder: patients with non-recognized affective or neurotic disorders and patients treated only as out-patients or in general practice were not included.

The proportion of women with neurotic disorders or non-psychotic affective disorders was substantially reduced during the study period because the number of available beds in psychiatric departments and hospitals was reduced by more than 50%. Since no differences in breast cancer risk were observed during the study period by calendar year at cohort entry, we have no reason to believe that cases with neurotic disorders or non-psychotic affective disorders have more risk than cases with psychotic affective disorders.

We suppose that the low frequency of alcohol abuse (8%) was due to the well-known tendency to underreport this stigmatizing diagnosis in women during the actual study period. Non-differential misclassification could, therefore, be the reason that alcohol abuse was not a significant risk factor in this cohort, in contrast to studies of females with alcohol abuse (Smith-Warner, 1998). Similarly, unspecified alcohol/drug abuse was not a risk factor in this cohort. We found no evidence of a positive relationship between length of admission and breast cancer risk, and indeed the trend with increased admission-length was negative.

Women admitted with psychiatric disorders have a higher prevalence of chronic medical illness (Fink, 1992) compared to the general population of women, implying a higher use of the health care system and increased possibility of having breast cancer diagnosed, which could have induced a slight overestimation. In this Danish nationwide cohort study with good clinical case identification, large sample size and low loss of follow-up, women discharged from psychiatric departments and hospitals with affective or neurotic disorders have the same incidence of breast cancer as women in the general population, adjusted for age and calendar period.

### ACKNOWLEDGEMENTS

We thank Professor Thorkil Sørensen, University Hospital of Odense, for counselling on the categorization of types of the diagnoses, Gurli Perto system consultant and Andrea Bautz system consultant for computer assistance. This study was supported by a grant from The Danish Cancer Society, Bispebjerg Hospital, Copenhagen Hospital Co-operation, and The Foundation of Martha and Axel Thomsen. Dr Mortensen received a grant from the Theodore and Vada Stanley Foundation. The study was approved by the local Ethics Committee (KA 95142 g). There was no conflict of interest.

### REFERENCES

Andersen BL, Farrar WB, Golden-Kreutz D, Kutz LA, MacCallum R, Courtney ME and Glaser R (1998) Stress and immune responses after surgical treatment for regional breast cancer. *J Natl Cancer Inst* **90**: 30–36

Bleiker EMA, Ploeg HM, Hendriks JHCL and Ader HJ (1996) Personality factors and breast cancer development: a prospective longitudinal study. *J Natl Cancer Inst* **88**: 1478–1482

Chen CC, David AS, Nunnerley H, Michell M, Dawson JL, Berry H, Dobbs J and Fahy T (1995) Adverse life events and breast cancer: case control study. *Br Med J* **311**: 1527–1530

Clayton D and Hills M (1993) *Statistical Models in Epidemiology*. Oxford University Press: New York

Cohen S and Rabin BS (1998) Psychologic stress, immunity, and cancer. *J Natl Cancer Inst* **90**: editorials 3–4

Danish National Board of Health (1997) *Cancer Incidence in Denmark 1994, Vital Statistics 1997*: 7. Danish National Board of Health: Copenhagen

Ewertz M (1986) Bereavement and breast cancer. *Br J Cancer* **53**: 701–703

Fink P (1992) *Psychiatric and Somatic Comorbidity* (in Danish). Institute for Basic Psychiatric Research: Psychiatric Hospital, Risskov

Ginsberg A, Price S, Ingram D and Nottage E (1996) Life events and the risk of breast cancer: a case–control study. *Eur J Cancer* **32A**: 2049–2052

Hagnell O (1966) The premorbid personality of persons who develop cancer in a total population investigated in 1947 and 1957. *Ann NY Acad Sci* **125**: 846–855

Hahn RC and Petitti DB (1988) Minnesota multiphasic personality inventory-rated depression and the incidence of breast cancer. *Cancer* **61**: 845–848

Irwin M, Smith TL and Gillin JC (1987) Low natural killer cytotoxicity in major depression. *Life Sci* **41**: 2127–2133

Johansen C and Olsen JH (1997) Psychological stress, cancer incidence and mortality from non-malignant diseases. *Br J Cancer* **75**: 144–148

Jones DR, Goldblatt PO and Leon DA (1984) Bereavement and cancer: some data on deaths of spouses from the longitudinal study of Office of Population Censuses and Surveys. *Br Med J* **289**: 461–464

Kessing LV (1998) Validity of diagnoses and other clinical register data in patient with affective disorder. *Eur J Psychiatry* (in press)

Kvikstad A, Vatten LJ, Tretli S and Kvinnsland S (1994) Death of a husband or marital divorce related to risk of breast cancer in middle-aged women. A nested case-control study among Norwegian women born 1935–1954. *Eur J Cancer* **30A**: 473–477

Maes M, Stevens W, Peeters D, DeClerk L, Scharpe S, Bridts C, Schotte C and Cosyns P (1992) A study on the blunted natural killer cell activity in severely depressed patients. *Life Sci* **50**: 503–513

Munk-Jørgensen P and Mortensen PB (1997) The Danish Psychiatric Central Register. *Danish Med Bull* **44**: 82–84

Roberts FD, Newcomb PA, Trentham-Dietz A and Storer BA (1996) Self-reported stress and risk of breast cancer. *Cancer* **77**: 1089–1093

Rostgaard K and Lynge E (1997) Incidence and mortality of breast cancer since 1950 (in Danish). *Danish Breast Cancer Cooperative Group* **5**: 14–18

 *© 1999 Cancer Research Campaign*

Schleifer SJ, Keller SE, Bartlett JA, Eckholdt HM and Delaney BR (1996) Immunity in young adults with major depressive disorder. *Am J Psychiatry* **153**: 477–482

Smith-Warner SA, Spiegelman D, Shiaw-Shyuan Y, van den Brandt PA, Folsom AR, Goldohm A, Graham S, Holmberg L, Howe GR, Marshall JR, Miller AB, Potter JD, Speizer FE, Wilett WC, Wolk A and Hunter DJ (1998) Alcohol and breast cancer in women. *JAMA* **279**: 535–540

Statistics Denmark (1998): Copenhagen

Steingart AB and Cotterchio M (1995) Do antidepressants cause, promote, or inhibit cancers? *J Clin Epidemiol* **48**: 1407–1412

Storm HH (1977) *Validity of Death Certificates for Cancer Patients in Denmark*. Danish Cancer Society: Copenhagen

Storm HH (1988) Completeness of cancer registration in Denmark 1943–1966 and efficacy of record linkages procedures. *Int J Epidemiol* **17**: 44–49

Storm HH, Pihl J and Michelsen AL (1996) *Cancer Incidence in Denmark 1993*. Danish Cancer Society: Copenhagen

Weeke A and Væth M (1986) Excess mortality of bipolar and unipolar manic-depressive patients. *J Affect Disord* **11**: 227–234

World Health Organization (1967) *Manual of the International Classification of Diseases, Injuries and Causes of Death. Based on the Recommendations of the Eighth Revision Conference, 1965, and Adopted by the Nineteenth World Health Assembly*. World Health Organization: Geneva

Zilber N, Schufman N and Lerner Y (1989) Mortality among psychiatric patients: the groups at risk. *Acta Psychiatry Scand* **79**: 248–256

Zonderman AB, Costa PT and McCrae RR (1989) Depression as a risk for cancer morbidity and mortality in a nationally representative sample. *JAMA* **262**: 1191–1195

Ødegaard Ø (1980) Fertility of psychiatric first admissions in Norway 1936–1975. *Acta Psychiatry Scand* **62**: 212–220

*© 1999 Cancer Research Campaign*

## References

[PDF_00379] Andersen B. L., Farrar W. B., Golden-Kreutz D., Kutz L. A., MacCallum R., Courtney M. E., Glaser R. (1998). Stress and immune responses after surgical treatment for regional breast cancer.. J Natl Cancer Inst.

[PDF_00382] Bleiker E. M., van der Ploeg H. M., Hendriks J. H., Adèr H. J. (1996). Personality factors and breast cancer development: a prospective longitudinal study.. J Natl Cancer Inst.

[PDF_00385] Chen C. C., David A. S., Nunnerley H., Michell M., Dawson J. L., Berry H., Dobbs J., Fahy T. (1995). Adverse life events and breast cancer: case-control study.. BMJ.

[PDF_00394] Ewertz M. (1986). Bereavement and breast cancer.. Br J Cancer.

[PDF_00399] Hagnell O. (1966). The premorbid personality of persons who develop cancer in a total population investigated in 1947 and 1957.. Ann N Y Acad Sci.

[PDF_00401] Hahn R. C., Petitti D. B. (1988). Minnesota Multiphasic Personality Inventory-rated depression and the incidence of breast cancer.. Cancer.

[PDF_00403] Irwin M., Smith T. L., Gillin J. C. (1987). Low natural killer cytotoxicity in major depression.. Life Sci.

[PDF_00405] Johansen C., Olsen J. H. (1997). Psychological stress, cancer incidence and mortality from non-malignant diseases.. Br J Cancer.

[PDF_00407] Jones D. R., Goldblatt P. O., Leon D. A. (1984). Bereavement and cancer: some data on deaths of spouses from the longitudinal study of Office of Population Censuses and Surveys.. Br Med J (Clin Res Ed).

[PDF_00412] Kvikstad A., Vatten L. J., Tretli S., Kvinnsland S. (1994). Death of a husband or marital divorce related to risk of breast cancer in middle-aged women. A nested case-control study among Norwegian women born 1935-1954.. Eur J Cancer.

[PDF_00416] Maes M., Stevens W., Peeters D., DeClerck L., Scharpe S., Bridts C., Schotte C., Cosyns P. (1992). A study on the blunted natural killer cell activity in severely depressed patients.. Life Sci.

[PDF_00419] Munk-Jørgensen P., Mortensen P. B. (1997). The Danish Psychiatric Central Register.. Dan Med Bull.

[PDF_00421] Roberts F. D., Newcomb P. A., Trentham-Dietz A., Storer B. E. (1996). Self-reported stress and risk of breast cancer.. Cancer.

[PDF_00427] Schleifer S. J., Keller S. E., Bartlett J. A., Eckholdt H. M., Delaney B. R. (1996). Immunity in young adults with major depressive disorder.. Am J Psychiatry.

[PDF_00429] Smith-Warner S. A., Spiegelman D., Yaun S. S., van den Brandt P. A., Folsom A. R., Goldbohm R. A., Graham S., Holmberg L., Howe G. R., Marshall J. R. (1998). Alcohol and breast cancer in women: a pooled analysis of cohort studies.. JAMA.

[PDF_00434] Steingart A. B., Cotterchio M. (1995). Do antidepressants cause, promote, or inhibit cancers?. J Clin Epidemiol.

[PDF_00436] Storm H. H. (1988). Completeness of cancer registration in Denmark 1943-1966 and efficacy of record linkage procedures.. Int J Epidemiol.

[PDF_00448] Zilber N., Schufman N., Lerner Y. (1989). Mortality among psychiatric patients--the groups at risk.. Acta Psychiatr Scand.

[PDF_00450] Zonderman A. B., Costa P. T., McCrae R. R. (1989). Depression as a risk for cancer morbidity and mortality in a nationally representative sample.. JAMA.

